# The Anti-Arthritic Efficacy of Khellin Loaded in Ascorbyl Decanoate Nanovesicles after an Intra-Articular Administration

**DOI:** 10.3390/pharmaceutics13081275

**Published:** 2021-08-17

**Authors:** Giulia Vanti, Lorenzo Di Cesare Mannelli, Laura Micheli, Lorenzo Cinci, Lucia Grifoni, Maria Camilla Bergonzi, Carla Ghelardini, Anna Rita Bilia

**Affiliations:** 1 Department of Chemistry Ugo Schiff, University of Florence, Via Ugo Schiff 6, Sesto Fiorentino, 50019 Florence, Italy; giulia.vanti@unifi.it (G.V.); lucia.grifoni@stud.unifi.it (L.G.); mc.bergonzi@unifi.it (M.C.B.); 2 Department of Neuroscience, Psychology, Drug Research and Child Health (NEUROFARBA), Pharmacology and Toxicology Section, Università degli Studi di Firenze, Viale G. Pieraccini 6, 50139 Florence, Italy; lorenzo.mannelli@unifi.it (L.D.C.M.); laura.micheli@unifi.it (L.M.); lorenzo.cinci@unifi.it (L.C.); carla.ghelardini@unifi.it (C.G.)

**Keywords:** nanovesicles, ascorbyl decanoate, khellin, osteoarthritis, MIA, pain, intra-articular treatment

## Abstract

Osteoarthritis is the most widespread joint-affecting disease. The management of persistent pain remains inadequate and demands new therapeutic strategies. In this study, we explored the pain relieving and protective properties of a single intra-articular (i.a.) injection of khellin loaded in nanovesicles (K-Ves) based on ascorbyl decanoate plus phosphatidylcholine in a rat model of osteoarthritis (OA) induced by monosodium iodoacetate (MIA) treatment. The developed nanovesicles (approximately 136 nm) had a narrow size distribution (PdI 0.26), a good recovery (about 80%) and a worthy encapsulation efficiency (about 70%) with a ζ-potential of about −40 mV. The stability of K-Ves was assessed in simulated synovial fluid. Seven days after the articular damage with MIA, both K-Ves and a suspension of khellin (K, 50 μL) were i.a. injected. K-Ves significantly counteracted MIA-induced hypersensitivity to mechanical noxious (paw pressure test) and non-noxious stimuli (von Frey test) and significantly reduced the postural unbalance related to spontaneous pain (incapacitance test) and the motor alterations (beam balance test) 7 and 14 days after the i.a. injection. K was partially active only on day 7 after the treatment. The histology emphasized the improvement of several morphological factors in MIA plus K-Ves-treated animals. In conclusion, K-Ves could be successfully used for the local treatment of osteoarthritis.

## 1. Introduction

Osteoarthritis (OA) refers to a clinical syndrome of joint pain accompanied by varying degrees of functional limitation and a reduced quality of life. It is the most common form of arthritis and one of the leading causes of pain and disability worldwide, characterized by the gradual development of mutable joint pain, swelling and difficulty and loss of motion [1,2]. Different therapeutic interventions are usually carried out [3], all with the aim to reduce pain and delay the pathology progression. Historically, OA was considered a mechanical disorder but it is increasingly recognized that local inflammation and damage at the joint level are important for the pathophysiology [4,5] and for the disease progression of OA becoming a main target for a therapeutic intervention.

Early-stage drug intervention can reduce the development of osteoarthritis and structurally and functionally repair the joints but the clinical efficacy of conventional therapeutic approaches is very poor, principally due to the formulations, which are not able to efficiently penetrate the cartilage tissues and deliver proper drug doses over time, determining minimal off target effects. Local intra-articular (i.a.) injections of drugs are generally performed to overcome the low joint bioavailability after a systemic administration. The majority of drugs administered intra-articularly are inappropriate because they quickly leave the synovial joint (generally half-lives are 1–4 h) and, as a consequence, numerous high drug dose injections are needed, producing toxicity. The rapid drug clearance from the joint represents the critical limit in the drug clinical efficacy for the majority of therapeutic interventions, as extensively highlighted in two recent reviews [6,7]. Significant effort has been focused on developing drug delivery systems to sustain drug levels within the joint for prolonged periods of time and, in particular, nanoscale drug carriers have been administered within the joint, which prolong the drug residence time within the joint and provide a sustained drug release intended for pain and inflammation relief with a single low-dose administration [8].

In this manuscript, we investigated the pain relief properties of a single i.a. injection of khellin-loaded nanovesicles (K-Ves) in a rat model of OA induced by an MIA injection. A comparison between a single i.a. injection of a standard khellin suspension was performed. We also carried out a histological analysis of the joint in order to highlight a possible protective effect of the formulation. Khellin is a natural furanochromone that has numerous interesting pharmacological properties related to anti-inflammatory activity. It has also been reported to be an EGFR inhibitor with an IC50 of 0.15 µM and consequent anti-proliferative activity as well as an inhibitor of AP-1 and NF-κB signals [9]. Lastly, we recently reported that alkanoyl-6-*O*-ascorbic acid esters, obtained upon the esterification of ascorbic acid’s primary hydroxyl groups with fatty acids, can enhance the stability of the phosphatidylcholine bilayer of nanovesicles, retaining the properties of ascorbic acid [10]. Accordingly, ascorbyl decanoate was added to soybean phosphatidylcholine (P90G) to stabilize the hydrophobic region of the nanovesicles before khellin loading.

## 2. Materials and Methods

### 2.1. Materials

A commercial khellin (95% purity) was purchased from Galeno Srl (Comeana, Italy). Soybean phosphatidylcholine Phospholipon^®^ 90G (P90G) was purchased from Lipoid GmbH (Ludwigshafen, Germany) with the support of the Italian agency AVG Srl. Human serum albumin and phosphate buffer saline (PBS; 0.138 M NaCl and 0.0027 M KCl; pH 7.4) were purchased from Sigma Aldrich (Milan, Italy) and hyaluronic acid (molecular weight range of 40–3000 KDa) was obtained from Altergon Italia Srl (Morra De Sanctis, Avellino, Italy). All the solvents (Sigma Aldrich, Milan, Italy) were of HPLC grade. Water was purified by a Simplicity^®^ UV water purification system from Merck Life Sciences Srl (Milan, Italy) and phosphotungstic acid (PTA) was purchased from Electron Microscopy Sciences (Hatfield, Pennsylvania, USA). 6-*O*-decanoyl-L-ascorbic acid (ascorbyl decanoate) was not a commercial sample and it was synthesized and purified with a final yield of 50%, as previously reported [11].

### 2.2. Preparation of the Nanovesicles

The nanovesicles were prepared by the film hydration method as previously reported [10,12]. Preliminary experiments were performed to verify the nanovesicle formation using different mole ratios of ascorbyl decanoate and P90G, using a fixed amount of P90G (13.8 mg/mL) and an increasing amount of ascorbyl decanoate (from 3.3 mg/mL to 13.2 mg/mL). In brief, the lipid substances were solubilized in a proper amount of dichloromethane and the volatile solvent was evaporated under a vacuum (Rotavapor R200, Büchi Labortechnik AG, Flawil, Switzerland) in a water bath at 37 °C. The lipid film obtained after the evaporation was hydrated with 10 mL of ultrapure water and stirred at 850 rpm for 30 min in a water bath at 38 ± 2 °C. The dispersion was ultrasonicated using a Sonopuls HD 2200 ultrasound device (Bandelin Electronic GmbH, Berlin, Germany) coupled to an MS 72 probe. A sonication time of 1 min with pulsed duty cycles of 1/2 s on and 1/2 s off was applied with an amplitude of 50%. The same method was used to prepare K-Ves. Briefly, khellin (2 mg/mL) was added to P90G (13.8 mg/mL) and ascorbyl decanoate (13.2 mg/mL) and solubilized in dichloromethane; the solvent was evaporated to obtain the thin lipid film and the obtained nanovesicles were optimized by ultrasonication.

### 2.3. Dynamic Light Scattering (DLS) and Electrophoretic Light Scattering (ELS)

Dynamic light scattering (DLS) was used to determine the mean size and polydispersity (PDI) of the nanovesicles [11]. DLS was performed using a Zetasizer Nano series ZS90 apparatus (Malvern Instruments, Worcestershire, UK) equipped with a 4 mW He-Ne laser operating at 632.8 nm, an optical fiber-based detector and a digital LV/LSE-5003 correlator. Ten measurements were performed in triplicate at 25 ± 2 °C using an optical quality 10 × 10 × 45 mm polystyrol/polystyrene cell at 90°. A total of 20 µL of each sample was diluted to 1 mL using ultrapure water. The cumulant method was used with the aid of Zetasizer software version 7.02 (Malvern Panalytical Ltd., Almelo, The Netherlands). Electrophoretic light scattering (ELS), using the same apparatus and the same operating conditions as DLS, gave the ζ-potential (mV) [13]. A Henry correction to Smoluchowski’s equation was used for the calculation of the electrophoretic mobility.

### 2.4. Morphological Characterization

The shape and morphology of the nanovesicles were evaluated by transmission electron microscopy (TEM) on a TEM CM12 PHILIPS equipped with an OLYMPUS Megaview G2 camera at an accelerating voltage of 80 keV. For the analyses, 10 µL samples were diluted at a ratio of 1:10 and placed on a carbon film-covered copper grid. Subsequently, most of the samples were blotted from the grid with filter paper to obtain a thin film that was stained with a phosphotungstic acid solution (1% *w/v*) in distilled water. The analysis of the samples was done 3 min after the staining [14].

### 2.5. HPLC-DAD Analyses

The quantification of the khellin uploaded in the nanovesicles was performed with a HP 1100 liquid chromatograph using a DAD detector and an HP 9000 workstation (Agilent Technologies Italia Spa, Italy). The column was 150 mm, 4.6 mm i.d. and 3.5 μm Eclipse XDB-C18 and the mobile phase was a gradient with a flow rate of 0.4 mL/min. The gradient was the following: (A) formic acid/water pH 3.2 and (B) CH_3_CN using the following gradient: 0–13 min: 50–80% B; 13–15: min 80–50% B; 15–18: min 50–50% B; with a post-time of 5 min. The analyses were performed at 27 ± 2 °C, recording the UV-Vis spectra in the 200–600 nm range and acquiring the spectra at 246 nm. Khellin gave a linear correlation (y = 0.00004144x − 0.00313111; R^2^ ≥ 0.9999) in the range from 0.005 to 1.00 µg.

### 2.6. Encapsulation Efficiency and Recovery

The encapsulation efficiency (EE%) of khellin was evaluated using a direct method [15] by the dialysis bag method. Briefly, 2 mL of nanovesicles were put into a Spectra/Por^®^ dialysis tubing of regenerated cellulose a with molecular weight cut-off of 3.5 KDa (Repligen Europe B.V., Breda, The Netherlands). The dialysis was performed using 1 L of purified water for 1 h under stirring. For the analysis, 100 µL of the purified sample was diluted with 900 µL ethanol and submitted to ultrasonication for 15 min and ultracentrifugation for 10 min at 14,000 rpm. The obtained supernatant was evaluated by HPLC-DAD as described in Section 2.5. The following formula was used for the calculation of *EE*% (1):

(1) $$EE\left(\%\right)=100\%\times \frac{encapsulated\left(mg/mL\right)}{weighted\;drug\;\left(mg/mL\right)}$$

In addition, the recovery (*R*%) of khellin in the formulation, defined as the percentage of the total drug recovered after the preparation procedure in relation to the weighed drug, was determined using the same procedure as EE% without the purification step by dialysis and it was calculated by Equation (2):

(2) $$R\left(\%\right)=100\%\times \frac{total\;recovered\;drug\left(mg/mL\right)}{weighted\;drug\;\left(mg/mL\right)}$$

### 2.7. Stability Studies in Simulated Synovial Fluid

A simulated synovial fluid (SSF) was prepared as reported in the literature [16] by solubilizing 3 g/L hyaluronic acid (HA), 19 g/L human serum albumin (HSA), 11 g/L IgG and 0.1 g/L P90G in PBS. K-Ves were diluted in freshly prepared SSF in a 1:1 volumetric ratio and were incubated at the controlled temperature of 37 °C in the dark, applying a horizontal mechanic stirring at 300 rpm by a Biosan PST-60HL thermo-shaker (Riga, Latvia). The stability test was carried out for two weeks according to the duration of the in vivo studies. Every other day, the size, PDI and ζ-potential were investigated by DLS/ELS. Eventual color changes and precipitation processes were also assessed.

### 2.8. Animals

Male Sprague–Dawley rats (Envigo, Varese, Italy) weighing approximately 200–250 g at the beginning of the experiments were used for all the analyses described below. Animals were housed at CeSAL (Centro Stabulazione Animali da Laboratorio, University of Florence) and used for the experiments at least one week after their arrival. For each cage, four rats were housed (size 26 × 41 cm^2^). The animals were preserved at 23 ± 1 °C using a 12 h light/dark cycle. They were fed with an ordinary diet and tap water ad libitum at 7:00 a.m. The studies were undertaken according to Directive 2010/63/EU, the European Union legislation that protects animals being used in research. In addition, the University of Florence ethical policy fulfills the Guide for the Care and Use of Laboratory Animals of the US National Institutes of Health (NIH Publication No. 85–23, revised 1996; University of Florence assurance number: A5278-01). Animal pain and the number of animals used were minimized. Furthermore, the Italian Ministry of Health (No. 498/2017-PR) and the Animal Subjects Review Board of the University of Florence gave the official endorsements to carry out the experiments. The studies on animals were performed in agreement with ARRIVE guidelines [17].

### 2.9. MIA-Induced Osteoarthritis

A monoiodoacetate (MIA, Sigma-Aldrich, Milan, Italy) injection was used to induce unilateral osteoarthritis into the tibiotarsal joint according to [18,19]. A total of 2% isoflurane was used to anesthetize the rats and the left leg skin was sterilized with 75% ethyl alcohol. The lateral malleolus was found by palpation and a 28-gauge needle was introduced perpendicularly into the cavity between the tibio-fibular and tarsal bone until a distinctive loss of opposition was found. MIA (2 mg/25 μL of saline) was injected monolaterally (ipsilateral paw). The rats of the control group were injected with a saline solution.

### 2.10. Treatments

Nanovesicles (Ves), K-Ves (2 mg/mL) and a suspension of khellin (K, 0.3 mg/mL) were injected into the tibiotarsal joint 7 days after the articular damage induced by MIA (day 1). As previously described for the MIA injection, the animals were anesthetized by 2% isoflurane and the ipsilateral paw was sterilized with 75% ethyl alcohol. The lateral malleolus was then found by palpation and a 28-gauge needle was inserted perpendicularly and rotated distally into the articular space of the cavity between the tibiofibular and tarsal bone. A quantity of 50 μL of formulation was then injected (day 1) [20]. The rats of the control group received the same volume of a saline solution. Behavioral studies were performed 7 and 14 days after the treatments.

### 2.11. Paw Pressure Test

The mechanical hyperalgesia of rats was determined by an analgesimeter (Ugo Basile, Varese, Italy) according to the methods described by [21,22]. Briefly, a regular growing weight was applied to a limited area of the dorsal surface of the hind paw using a blunt conical probe by a mechanical device. The mechanical pressure (expressed in grams) was increased until vocalization or a withdrawal reflex happened while rats were lightly restrained. A cut-off value of 100 g was adopted.

### 2.12. Von Frey Test

The mechanical allodynia was measured with an electronic von Frey hair unit (Ugo Basile, Varese, Italy). Briefly, the rats were located in 20 × 20 cm Plexiglas boxes furnished with a metallic mesh floor 20 cm above the bench. After 15 min of adaptation, a force ranging from 0 to 50 g (accuracy of 0.2 g) was applied to estimate the withdrawal threshold. A punctuate stimulus was applied to the mid-plantar area of each anterior paw from below the mesh floor through a plastic tip and the withdrawal threshold was automatically displayed on the screen. The paw sensitivity threshold was defined as the lowest pressure necessary to provoke a vigorous and instantaneous withdrawal reflex of the paw. The measure was repeated 5 times with an interval of 5 s and then expressed as a mean [23,24].

### 2.13. Incapacitance Test

Spontaneous pain was evaluated by an incapacitance apparatus (Linton Instrumentation, Norfolk, UK) in order to record variations in the postural equilibrium [25,26]. The rats were trained to stand on their hind paws in a box. This box was located above the incapacitance apparatus, allowing us to autonomously measure the weight that the rat applied on each hind limb. The data represent the mean of five successive measurements for each animal. An unequal distribution of weight on the hind limbs showed a monolateral reduced pain threshold [18]. Data were articulated as the difference between the weight applied to the limb contralateral to the injury and the weight applied to the ipsilateral one (Δ weight).

### 2.14. Beam Balance Test

Motor coordination was evaluated by the beam balance test according to literature [27]. The test was done with the rats being placed on a narrow strip of wood (30 cm × 1.3 cm) while balancing and the scoring standards were as follows: 0 point, the four limbs were all on the wood in a balance situation; 1 point, the limbs of one side were able to grasp the wood or shake on the wood; 2 points, one or two limbs slipped from the wood; 3 points, three limbs slipped from the wood; 4 points, the rat was suspended on the wood and fell over after a struggle [20].

### 2.15. Histological Studies

The animals were sacrificed and the legs were collected and fixed in 4% formaldehyde in phosphate-buffered saline (PBS) for 48 h at room temperature for the histological studies. The samples were then decalcified using a 0.76 M sodium formate and 1.6 M formic acid solution in H_2_O for 4 weeks. The solution was changed every 7 days. The histological samples were firstly dehydrated in alcohol and then embedded in paraffin. Six μM sections were observed, attributing a histological score (0: absent; 1: mild; 2: moderate; 3: severe) following these morphological parameters: (a) inflammatory infiltrate; (b) synovial hyperplasia; (c) fibrin deposition; (d) synovial vascularity; (e) cartilage erosion; (f) bone erosion; (g) joint space [28,29].

### 2.16. Statistical Analysis

The researchers were blind to all treatments. The reported values represent the mean ± SEM of six rats per group, performed in two different experimental sets. The examination of variance was performed by an ANOVA. A Bonferroni’s significant difference procedure was used as a post-hoc comparison. *P*-values of less than 0.05 were considered significant. Data were analyzed using Origin 9 software (OriginLab, Northampton, MA, USA).

## 3. Results and Discussion

### 3.1. Formulation and Characterization of Nanovesicles

The lipid film hydration method was used for the preparation of the nanovesicles obtaining multilamellar structures. The development of nanovesicles was done using a fixed amount of P90G (13.8 mg/mL) and increasing concentrations of ascorbyl decanoate (from 3.3 mg/mL to 13.2 mg/mL). Growing amounts of ascorbyl decanoate reduced the size and polydispersity of the nanovesicles (see Table 1) suggesting a stabilizing effect of the ascorbyl derivative, also determined by the increasing value of the ζ-potential, which was linked to the deprotonation of the ascorbyl head-groups. The homogeneity was greatly increased by ultrasonication without an effect on the lipid bilayer of the nanovesicles. The optimization of both size and PDI was achieved using cycles of 1/2 s on and 1/2 s off. Based on these results, the formulation containing 13.2 mg/mL ascorbyl decanoate was selected for further investigations because of the small size, optimal for enhanced permeation and distribution, together with a little PDI in order to achieve constant in vivo performances and obtain a successful therapy. Finally, the value of the ζ-potential was ideal in terms of the stability of the nanovesicles, avoiding the phenomena of aggregation due to the high electrostatic repulsion. Therefore, the optimization of size, PDI and ζ-potential represent crucial issues for the development of effective nanocarriers. Higher amounts of ascorbyl decanoate gave the worst results in terms of the PDI, size and ζ-potential, probably due to a mixture of micelles and nanovesicles.

Nanovesicles prepared with 13.8 mg/mL of P90G and 13.2 mg/mL of ascorbyl decanoate were loaded with khellin (2 mg/mL) as briefly reported in the experimental section to obtain K-Ves. DLS and ELS analyses evidenced that khellin slightly modified the size of the nanovesicles by about 10 nm (from 128.7 ± 2.0 to 135.6 ± 7.2 nm) whereas the PDI remained in the same range (0.27 ± 0.01 versus 0.26 ± 0.03). The ζ-potential only marginally increased (from −43.1 ± 1.3 to −39.4 ± 0.6 mV).

**Figure 1:**
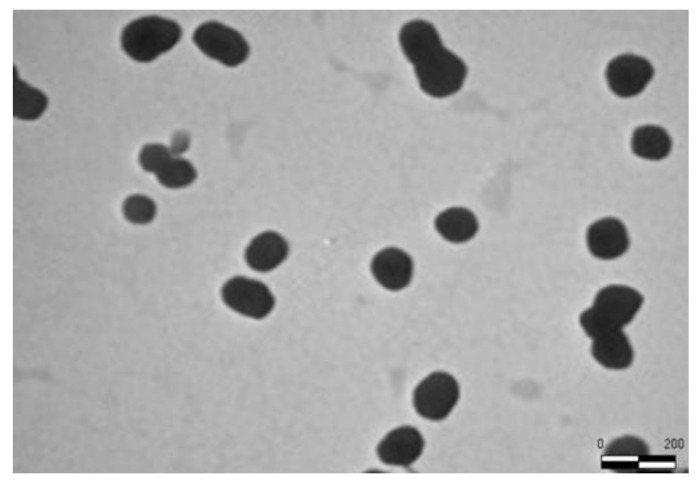
TEM image of K-Ves dispersion.

A TEM analysis of the optimized K-Ves dispersion was performed to further confirm the DLS analysis. A microscopic observation evidenced a nearly spherical shape of the nanovesicles with an average size similar to that obtained by DLS for pure K-Ves (Figure 1).

### 3.2. Khellin R% and EE%

Crucial properties of the developed nanovesicles were the *EE*% and *R*%, which were investigated by an HPLC-DAD analysis. Increasing the amount of khellin up to 2 mg/mL resulted in the highest *EE*% (67.96 ± 4.79) and R% (79.48 ± 2.21%). The results were obtained as an average of the triplicate measurements with a standard deviation. By contrast, amounts of khellin greater than 2 mg/mL resulted in a decrease in the drug loading, indicating that khellin imparted a negligible effect on the nanovesicles’ bilayer stability. The developed K-Ves formulations appeared to have optimal structural characteristics as drug delivery systems suitable for all types of administration.

### 3.3. Stability Studies of K-Ves in Simulated Synovial Fluid

The physical stability of K-Ves in SSF was investigated. An artificial liquid, with viscosity and lubrification properties similar to those of real synovial fluid, was prepared dissolving HA, HSA, IgG and P90G in PBS in a stepwise approach. The effects of the solutes on the nanovesicles were evaluated in terms of size, PDI and the ζ-potential, with an attempt to reproduce the synovial environment successively to a single i.a. injection of K-Ves. The stability was monitored for two weeks according to the timing of the in vivo studies because the animals were analyzed for two weeks after the administration of K-Ves. The nanovesicles were thereby incubated with SSF at 37 °C and analyzed every day for two weeks.

**Figure 2:**
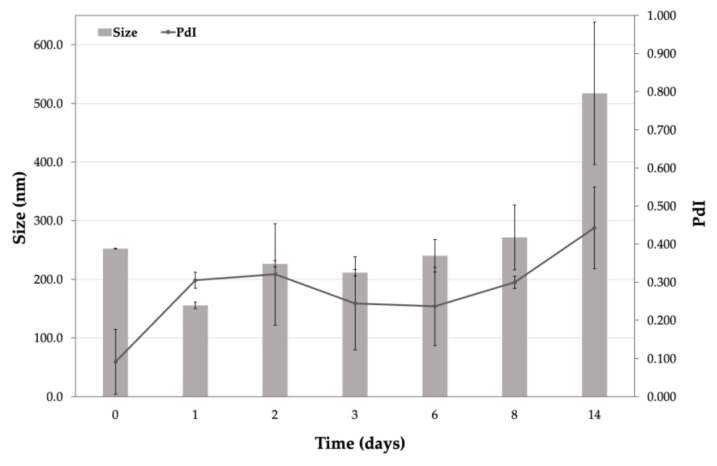
Size and PDI values of K-Ves during the stability test in simulated synovial fluid. Mean ± SD (*n =* 2).

**Figure 3:**
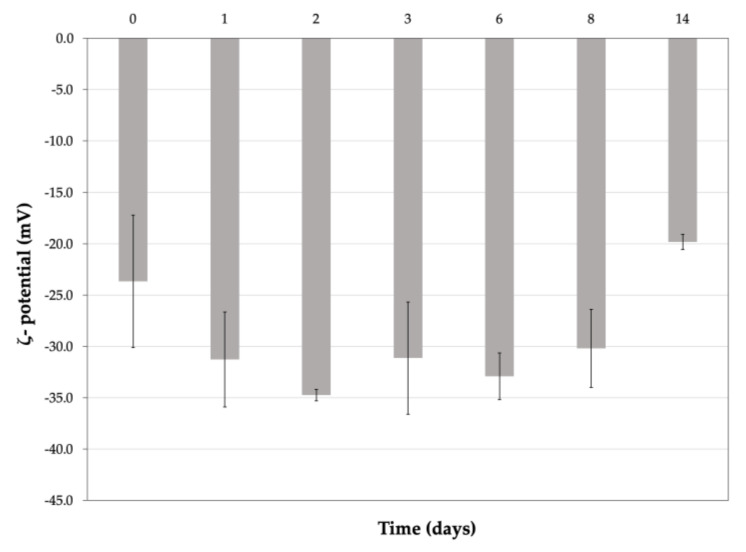
ζ-potential values of K-Ves during the stability test in simulated synovial fluid. Mean ± SD (*n=* 2).

In this study, we aimed to evidence the eventual aggregation, fusion or rupture of the vesicles. It was observed that the vesicles after dissolution in SSF increased the values of the size and PDI (Figure 2), probably due to the effects of the colligative properties, but neither the aggregation of the nanovesicles nor their rupture was observed. The ζ-potential was quite stable during the first eight days and then it decreased slowly but remained moderately negative (Figure 3). The results obtained by DLS were not statistically significant (excluding a few measurements) but were sufficient to assess that the simulated synovial fluid did not cause a rupture of the nanovesicles and that K-Ves could represent a possible successful formulation for an i.a. administration.

### 3.4. Behavioral Evaluation

**Figure 4:**
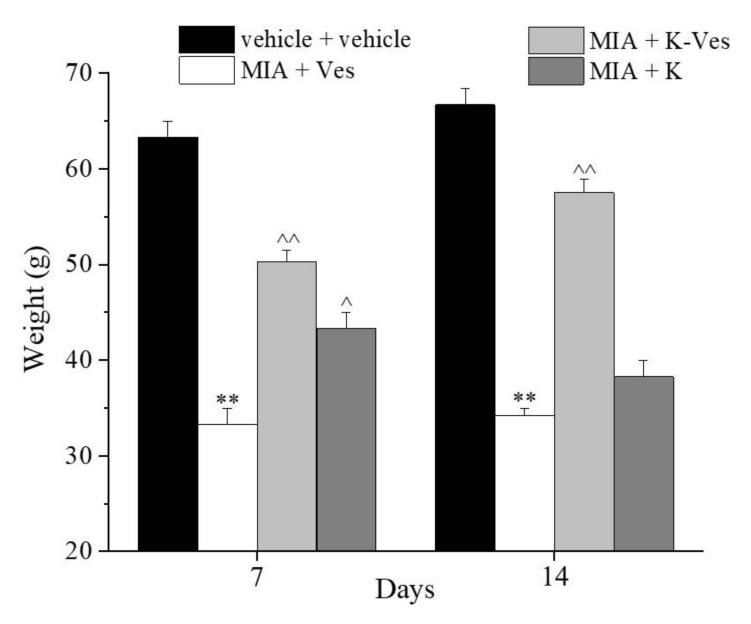
Articular pain related to mechanical hyperalgesia. Monoarthritis was induced by an MIA (2 mg/mL) injection into the tibio-tarsal joint (day 7). Fifty μL of Ves, K-Ves (2 mg/mL) or K (0.3 mg/mL) were i.a. administered 7 days after MIA (day 1). A paw pressure test was performed on day 7 and 14 after the treatments. Each value represents the mean ± SEM of six rats per group performed in two different experimental sets. ** *P* < 0.01 versus the vehicle + vehicle group; ^ *P* < 0.05 and ^^ *P* < 0.01 versus the MIA + Ves group.

**Figure 5:**
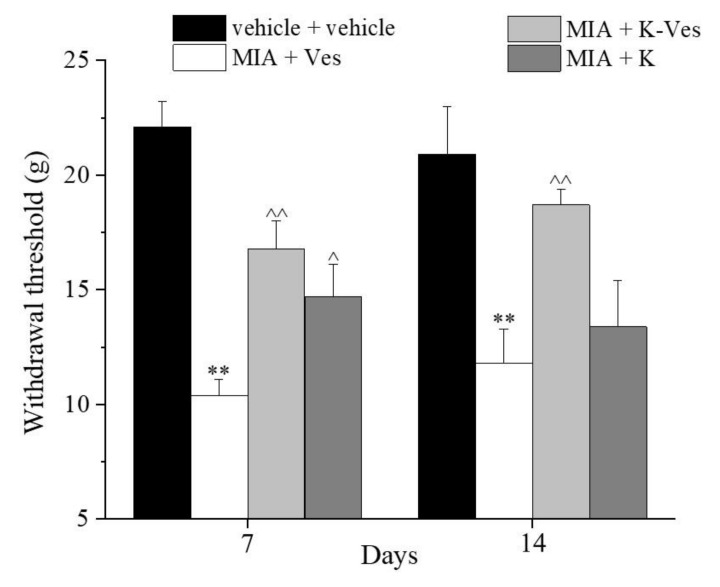
Articular pain related to mechanical allodynia. Monoarthritis was induced by an MIA (2 mg/mL) injection into the tibio-tarsal joint (day 7). Fifty μL of Ves, K-Ves (2 mg/mL) or K (0.3 mg/mL) were i.a. administered 7 days after MIA (day 1). A von Frey test was performed on day 7 and 14 after the treatments. Each value represents the mean ± SEM of six rats per group performed in two different experimental sets. ** *P* < 0.01 versus the vehicle + vehicle group; ^ *P* < 0.05 and ^^ *P* < 0.01 versus the MIA + Ves group.

**Figure 6:**
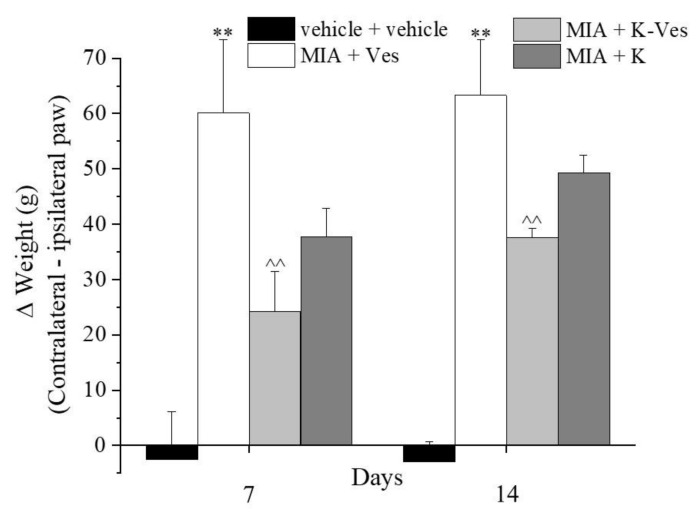
Articular spontaneous pain. Monoarthritis was induced by an MIA (2 mg/mL) injection into the tibio-tarsal joint (day 7). Fifty μL of Ves, K-Ves (2 mg/mL) or K (0.3 mg/mL) were i.a. administered 7 days after MIA (day 1). An incapacitance test was performed on day 7 and 14 after the treatments. Each value represents the mean ± SEM of six rats per group performed in two different experimental sets. ** *P* < 0.01 versus the vehicle + vehicle group; ^^ *P* < 0.01 versus the MIA + Ves group.

**Figure 7:**
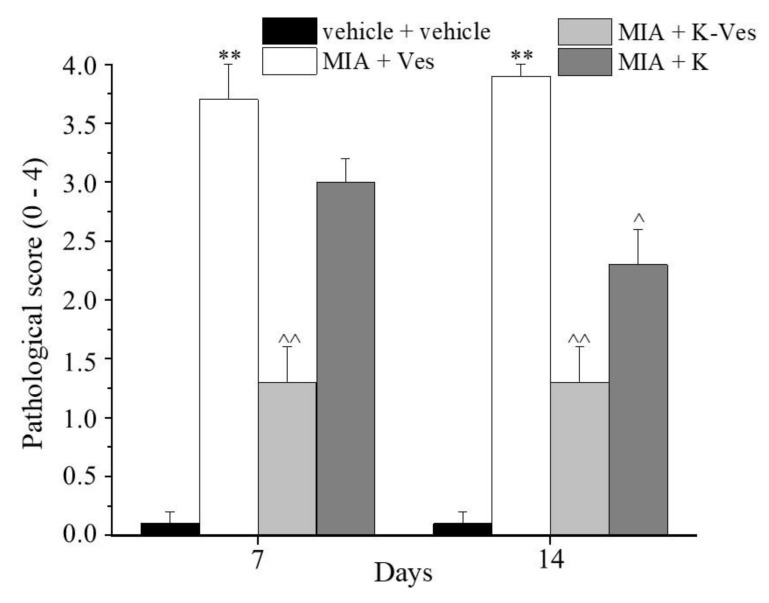
Motor abilities related to pain. Monoarthritis was induced by an MIA (2 mg/mL) injection into the tibio-tarsal joint (day 7). Fifty μL of Ves, K-Ves (2 mg/mL) or K (0.3 mg/mL) were i.a. administered 7 days after MIA (day 1). A beam balance test was performed on day 7 and 14 after the treatments. Each value represents the mean ± SEM of six rats per group performed in two different experimental sets. ** *P* < 0.01 versus the vehicle + vehicle group; ^ *P* < 0.05 and ^^ *P* < 0.01 versus the MIA + Ves group.

The effects of a single dose of K-Ves administered by i.a. were estimated using a rat model of unilateral osteoarthritis obtained by an MIA treatment. The efficacy of the K-Ves formulation was compared with the activity of a saturated khellin suspension named K. MIA is a chemical that inhibits glycolysis, disrupts the homeostatic balance of chondrocytes and evokes cartilage degeneration and subchondral bone loss mimicking human osteoarthritis [29,30]. The development of osteoarthritis pathology and pain in the MIA model typically occurs within 1–2 weeks following an i.a. MIA injection; the joint becomes hyperemic and edematous and there is an infiltration of circulating leucocytes [25]. By days 5–7, the inflammation subsides and remains at low levels throughout the subsequent development of MIA-induced OA [31]. The assessment of the pain threshold and motor skills of the animals was analyzed on day 7 and 14 after K-Ves or K injections; both formulations were injected only once when MIA-induced OA was well-established. At this time point, the response to a mechanical noxious stimulus was measured by the paw pressure test (Figure 4). The MIA treatment significantly decreased the pain threshold of the ipsilateral paw on both analyzed days with respect to the control group (vehicle plus vehicle). A K-Ves injection significantly improved the weight burdened on the posterior paw with an efficacy that increased over time. On the contrary, the K formulation was active only on day 7. These results highlight the importance of the khellin encapsulation for the long-lasting pain relief effect. The injection of the empty nanovesicles (MIA + Ves) was ineffective (Figure 4). Similar results were obtained with the evaluation of the mechanical allodynia assessed by the von Frey test (Figure 5). The K-Ves treatment increased the paw withdrawal threshold with respect to MIA + Ves-treated animals when they were stimulated by a non-noxious mechanical stimulus. The efficacy of the nanovesicles loaded with khellin was higher on day 7 with respect to MIA + K-treated animals and increased during the subsequent days and reached the maximum value on day 14. Unilateral pain induced by MIA was also able to evoke hind limb weight bearing alterations measured by the incapacitance test (Figure 6). This test is useful to analyze the treatment’s efficacy against spontaneous pain as no nociceptive or non-nociceptive stimuli were applied to the rat. The difference between the weight burdened on the contralateral paw and the ipsilateral one was significantly increased in the MIA + Ves group in comparison to the control animals both on day 7 and 14. K-Ves counteracted the spontaneous pain at all time points considered, showing a better profile in comparison to the K treatment that did not reach a statistical significance. Lastly, as shown in Figure 7, the MIA injection also provoked motor impairments and alterations evaluated by the beam balance test. The MIA-treated animals showed an increase in the pathological score with respect to the control group. K-Ves significantly improved the animal’s motor skills, halving the score assigned. The K injection was active only on day 14 but with a lower efficacy in comparison to the formulation of khellin in the nanovesicles. On day 14, the animals were sacrificed to perform the histological analysis of the tibio-tarsal joint. The MIA treatment evoked fibrin deposition in the joint space and several foci of cartilage and bone erosion, as illustrated in Figure 7.

### 3.5. Histological Evaluation of Synovia

**Figure 8:**
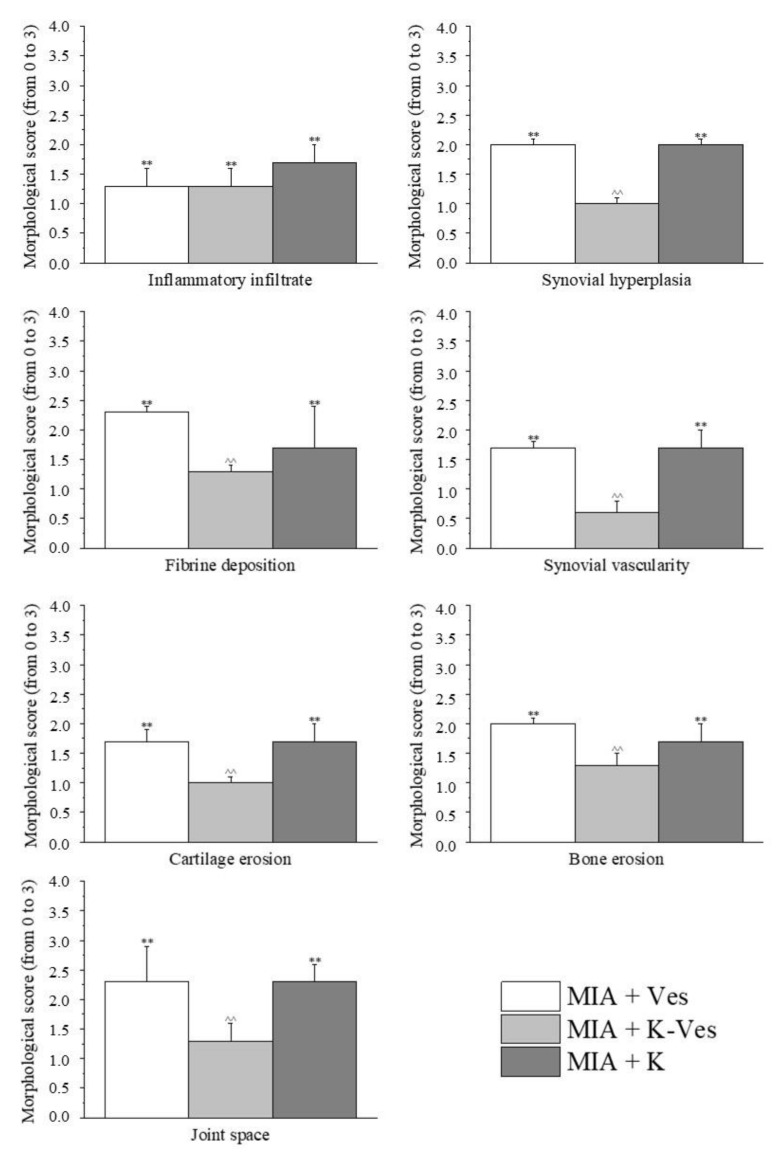
Morphological evaluations of the tibio-tarsal joints on day 14 after the treatments. The panel represents the quantification of the morphological parameters by a specific score (0: absent; 1: light; 2: moderate; 3: severe). The control animals all had a morphological score equal to 0 and are not reported in the graphs. Each value represents the mean ± SEM. of six rats per group performed in two different experimental sets. ** *P* < 0.01 versus the vehicle + vehicle group; ^^ *P* < 0.01 versus the MIA + Ves group.

**Figure 9:**
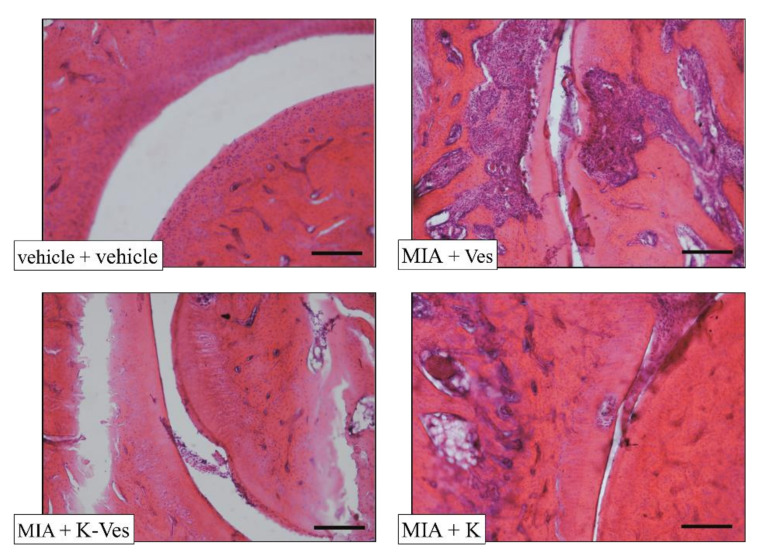
The panel shows the comparative images of the histological samples of the tibio-tarsal joints from each experimental group. Magnification: 100×; scale bar: 100 µM.

The histological evaluation of the synovia highlighted the presence of inflammatory infiltrate and synovial hyperplasia as well as an increased synovial vascularity (Figure 8 and Figure 9). A single K-Ves i.a. injection significantly reduced all parameters evaluated with the exception for the inflammatory infiltrate. The K treatment was ineffective, remarking once again the importance of the formulation of khellin in the nanovesicles. Of note, the control animals presented no signs of alteration, scoring 0 accordingly with previous results [32].

## 4. Conclusions

Natural products represent a unique chest of pleiotropic active molecules to promote health or to treat pathologies, especially complex diseases with a multifactorial etiology [33]. The main limitations to the successful clinical use of natural products are represented by their low water solubility and stability and poor biopharmaceutical characteristics, which can be overcome by different technological approaches, especially the development of nanocarriers [34].

Nanovesicles particularly have a great ability to allow a sustained and controlled release of the encapsulated drugs, resulting in an extraordinary therapeutic value [35]. This study focused on exploring the possibility of using ascorbyl decanoate as a nanovesicle bilayer-forming component and on evaluating the ability of this carrier to load khellin. Due to the narrow size distribution and adequate encapsulation efficiency of K-Ves together with a relative stability in simulated synovial fluid, these nanostructures were suitable for an i.a. injection. In particular, the efficacy of a single i.a. injection was highlighted in a rat model of MIA-induced osteoarthritis. In the in vivo tests, the K-Ves formulation was able to reduce articular pain, measured as evoked or spontaneous pain, as well as motor impairments. Moreover, a histological analysis of the joints highlighted the protective properties of K-Ves, suggesting this treatment as a new candidate for the management of articular pain. Further investigations may offer new insights into the pharmacodynamic profile of khellin as well as its gender-related efficacy.

## Figures and Tables

**Table 1:** Composition and characterization of the nanovesicles.

Ascorbyl Decanoate (mg/mL)	P90G (mg/mL)	PDI	Size (nm)	ζ-Potential (mV)
3.2	13.8	0.38 ± 0.02	214.7 ± 1.5	−36.2 ± 0.3
6.4	13.8	0.32 ± 0.02	172.8 ± 2.9	−44.2 ± 0.1
9.6	13.8	0.26 ± 0.01	134.0 ± 0.7	−44.5 ± 0.3
13.2	13.8	0.27 ± 0.01	128.7 ± 2.0	−43.1 ± 1.3

## Data Availability

The data presented in this study are available on request from the corresponding author.

## References

[1] Carr A.J. (1999). Beyond disability: Measuring the social and personal consequences of osteoarthritis. Osteoarthr. Cartil..

[2] Ma V.Y., Chan L., Carruthers K.J. (2014). Incidence, prevalence, costs, and impact on disability of common conditions requiring rehabilitation in the United States: Stroke, spinal cord injury, traumatic brain injury, multiple sclerosis, osteoarthritis, rheumatoid arthritis, limb loss, and back pain. Arch. Phys. Med. Rehabil..

[3] Sinusas K. (2012). Osteoarthritis: Diagnosis and treatment. Am. Fam. Physician.

[4] Benito M.J., Veale D.J., FitzGerald O., van den Berg W.B., Bresnihan B. (2005). Synovial tissue inflammation in early and late osteoarthritis. Ann. Rheum. Dis..

[5] Sellam J., Berenbaum F. (2010). The role of synovitis in pathophysiology and clinical symptoms of osteoarthritis. Nat. Rev. Rheumatol..

[6] Larsen C., Østergaard J., Larsen S.W., Jensen H., Jacobsen S., Lindegaard C., Andersen P.H. (2008). Intra-articular depot formulation principles: Role in the management of postoperative pain and arthritic disorders. J. Pharm. Sci..

[7] Evans C., Kraus V., Setton L. (2015). Progress in intra-articular therapy. Rheumatol. Nat. Rev..

[8] Mehtaa S., Hea T., Bajpayee A.G. (2021). Recent advances in targeted drug delivery for treatment of osteoarthritis. Curr. Opinion Rheumatol..

[9] Lee J.K., Jung J.S., Park S.H., Park S.H., Sim Y.B., Kim S.M., Ha T.S., Suh H.W. (2010). Anti-inflammatory effect of visnagin in lipopolysaccharide-stimulated BV-2 microglial cells. Arch. Pharm. Res..

[10] Risaliti L., Ambrosi M., Calamante M., Bergonzi M.C., Lo Nostro P., Bilia A.R. (2020). Preparation and characterization of ascosome vesicles loaded with khellin. J. Pharm. Sci..

[11] Capuzzi G., Lo Nostro P., Kulkarni K., Fernandez J.E. (1996). Mixtures of stearoyl-6-O-ascorbic acid and α-tocopherol: A monolayer study at the gas/water interface. Langmuir.

[12] Vanti G., Bani D., Salvatici M.C., Bergonzi M.C., Bilia A.R. (2019). Development and percutaneous permeation study of escinosomes, escin-based nanovesicles loaded with berberine chloride. Pharmaceutics.

[13] Vanti G., Coronnello M., Bani D., Mannini A., Bergonzi M.C., Bilia A.R. (2021). Co-delivery of berberine chloride and tariquidar in nanoliposomes enhanced intracellular berberine chloride in a doxorubicin-resistant K562 cell line due to P-gp overexpression. Pharmaceutics.

[14] Vanti G., Tomou E.M., Stojković D., Ćirić A., Bilia A.R., Skaltsa H. (2021). Nanovesicles loaded with *Origanum onites* and *Satureja thymbra* essential oils and their activity against food-borne pathogens and spoilage microorganisms. Molecules.

[15] Vanti G., Wang M., Bergonzi M.C., Zhidong L., Bilia A.R. (2020). Hydroxypropyl methylcellulose hydrogel of berberine chloride-loaded escinosomes: Dermal absorption and biocompatibility. Int. J. Biol. Macromol..

[16] Bortel E.L., Charbonnier B., Heuberger R. (2015). Development of a synthetic synovial fluid for tribological testing. Lubricants.

[17] McGrath J.C., Lilley E. (2015). Implementing guidelines on reporting research using animals (ARRIVE etc.): New requirements for publication in BJP. Br. J. Pharmacol..

[18] Di Cesare Mannelli L., Micheli L., Zanardelli M., Ghelardini C. (2013). Low dose native type II collagen prevents pain in a rat osteoarthritis model. BMC Musculoskelet. Disord.

[19] Maresca M., Micheli L., Cinci L., Bilia A.R., Ghelardini C., Di Cesare Mannelli L. (2017). Pain relieving and protective effects of Astragalus hydroalcoholic extract in rat arthritis models. J. Pharm. Pharmacol..

[20] Micheli L., Ghelardini C., Lucarini E., Parisio C., Trallori E., Cinci L., Di Cesare Mannelli L. (2019). Intra-articular mucilages: Behavioural and histological evaluations for a new model of articular pain. J. Pharm. Pharmacol..

[21] Leighton G.E., Rodriguez R.E., Hill R.G., Hughes J. (1988). κ-Opioid agonist produce antinociception after i.v. and i.c.v. but not intrathecal administration in the rat. Br. J. Pharmacol..

[22] Bird M.F., Cerlesi M.C., Brown M., Malfacini D., Vezzi V., Molinari P., Micheli L., Di Cesare Mannelli L., Ghelardini C., Guerrini R. (2016). Characterisation of the novel mixed mu-NOP peptide ligand dermorphin-N/OFQ (DeNo). PLoS ONE.

[23] Sakurai M., Egashira N., Kawashiri T., Yano T., Ikesue H., Oishi R. (2009). Oxaliplatin-induced neuropathy in the rat: Involvement of oxalate in cold hyperalgesia but not mechanical allodynia. Pain.

[24] Baptista-de-Souza D., Di Cesare Mannelli L., Zanardelli M., Micheli L., Nunes-de-Souza R.L., Canto-de-Souza A., Ghelardini C. (2014). Serotonergic modulation in neuropathy induced by oxaliplatin: Effect on the 5HT2C receptor. Eur. J. Pharmacol..

[25] Bove S.E., Calcaterra S.L., Brooker R.M., Huber C.M., Guzman R.E., Juneau P.L., Schrier D.J., Kilgore K.S. (2003). Weight bearing as a measure of disease progression and efficacy of anti-inflammatory compounds in a model of monosodium iodoacetate-induced osteoarthritis. Osteoarthr. Cartil..

[26] Maresca M., Micheli L., Di Cesare Mannelli L., Tenci B., Innocenti M., Khatib M., Mulinacci N., Ghelardini C. (2016). Acute effect of *Capparis spinosa* root extracts on rat articular pain. J. Ethnopharmacol..

[27] Ding Y., Li J., Lai Q., Rafols J.A., Luan X., Clark J., Diaz F.G. (2004). Motor balance and coordination training enhances functional outcome in rat with transient middle cerebral artery occlusion. Neuroscience.

[28] Snekhalatha U., Anburajan M., Venkatraman B., Menaka M. (2013). Evaluation of complete Freund’s adjuvant-induced arthritis in a Wistar rat model. Comparison of thermography and histopathology. Z. Rheumatol..

[29] Micheli L., Bozdag M., Akgul O., Carta F., Guccione C., Bergonzi M.C., Bilia A.R., Cinci L., Lucarini E., Parisio C. (2019). Pain relieving effect of-NSAIDs-CAIs hybrid molecules: Systemic and intra-articular treatments against rheumatoid arthritis. Int. J. Mol. Sci..

[30] Combe R., Bramwell S., Field M.J. (2004). The monosodium iodoacetate model of osteoarthritis: A model of chronic nociceptive pain in rats?. Neurosci. Lett..

[31] Guzman R.E., Evans M.G., Bove S., Morenko B., Kilgore K. (2003). Mono-iodoacetate-induced histologic changes in subchondral bone and articular cartilage of rat femorotibial joints: An animal model of osteoarthritis. Toxicol. Pathol..

[32] Micheli L., Di Cesare Mannelli L., Mattoli L., Tamimi S., Flamini E., Garetto S., Lucci J., Giovagnoni E., Cinci L., D’Ambrosio M. (2020). Intra-articular route for the system of molecules 14G1862 from *Centella asiatica*: Pain relieving and protective effects in a rat model of osteoarthritis. Nutrients..

[33] Bilia A.R., Piazzini V., Guccione C., Risaliti L., Asprea M., Capecchi G., Bergonzi M.C. (2017). Improving on Nature: The role of nanomedicine in the development of clinical natural drugs. Planta Med..

[34] Bilia A.R., Piazzini V., Risaliti L., Vanti G., Casamonti M., Wang M., Bergonzi M.C. (2019). Nanocarriers: A successful tool to increase solubility, stability and optimise bioefficacy of natural constituents. Curr. Med. Chem..

[35] FDA (2018). Liposome Drug Products; Chemistry Manufacturing and Controls; Human Pharmacokinetics and Bioavailability.

